# Time-Dependent Alterations in Walking Biomechanics After Anterior Cruciate Ligament Reconstruction Compared with Healthy Individuals: A Systematic Review with Meta-analysis

**DOI:** 10.1186/s40798-026-01016-x

**Published:** 2026-04-06

**Authors:** Ali Esmaeili, AmirAli Jafarnezhadgero, Heidar Sajedi, Elif Aydin, Tibor Hortobágyi, Urs Granacher

**Affiliations:** 1 Department of Sport Biomechanics, Faculty of Educational Science and Psychology, University of Mohaghegh Ardabili, Ardabil, Iran; 2 Sport Science Faculty, Department of Movement and Training Sciences, Düzce University, Düzce, Turkey; 3 Physical Education and Sports Department, Sport Science Faculty, Ardahan University, Ardahan, Turkey; 4 Department of Kinesiology, Hungarian University of Sports Science, Budapest, Hungary; 5 Faculty of Sport Science, Ningbo University, Ningbo, China; 6 Department of Human Movement Sciences, University Medical Center Groningen, University of Groningen, Groningen, Netherlands; 7 Department of Sport and Sport Science, Exercise and Human Movement Science, University of Freiburg, Freiburg, Germany

**Keywords:** Walking, Kinetics, Kinematics, ACL surgery

## Abstract

**Background:**

Knee injuries involving the anterior cruciate ligament (ACL) are often treated through surgical care to restore knee joint stability. Despite surgical treatment, there is evidence of altered biomechanical gait characteristics for short-, mid-, and even long-term post ACL reconstruction (ACLR).

**Objective:**

In this systematic review with meta-analysis, we aimed to analyze lower extremity joint kinematics and kinetics recorded during walking in individuals with ACLR and determine the time course of recovery of gait biomechanics following ACLR.

**Methods:**

Five electronic databases (Scopus, PubMed, EMBASE, Physiotherapy Evidence Database (PEDro), Cochrane Central Register of Controlled Trials [CENTRAL]) were systematically searched for articles potentially eligible for inclusion from inception until January 2026. A PECOS (Participants: ACLR individuals aged ≥ 18; Exposure: ACL surgery; Comparators: healthy controls; Outcomes: lower limb kinematics and kinetics during the walking stance phase; and Study design: case control studies, case series, cross-sectional studies, randomized controlled trials [baseline], cohort studies) approach was applied to define inclusion and exclusion criteria. Using cross-sectional studies, gait biomechanics were assessed short- (0–<6 months), mid- (≥ 6–12 months), and long-term (≥ 12 months) post-surgery in ACLR patients versus healthy controls. Gait biomechanics assessed during the stance phase were extracted from the included articles. Between-group standardized mean differences (SMD) with 95% confidence intervals (CI) were computed using a random-effects model to elucidate the gait biomechanical differences between ACLR patients and healthy controls. The modified version of the Downs and Black checklist was used to assess the methodological quality of the included studies.

**Results:**

The initial search identified 3199 hits and according to a priori defined in-/exclusion criteria, 31 cross-sectional studies with males and females aged 17–55 years were eligible to be included. The mean methodological quality of all included studies was moderate (Downs and Black checklist score: 68%). Based on outcomes from 17 studies, lower peak knee flexion angles were noted during the stance phase of walking in ACLR patients compared to controls (small SMD=-0.50, 95% CI -0.77 to -0.22, *p* = 0.0004, I^2^ = 79%) which could even be found ≥ 12 months post-surgery (11 studies: small SMD=-0.49, 95% CI -0.84 to -0.15, *p* = 0.005, I^2^ = 83%). Up until six months post-surgery, lower peak knee adduction angles (four studies: moderate SMD=-0.56, 95% CI -0.96 to -0.16, *p* = 0.006, I^2^ = 47%) were observed in ACLR patients. Regarding kinetics, irrespective of the time point post ACLR, the peak knee flexor joint moments (11 studies: moderate SMD=-0.57, 95% CI -0.90 to -0.25, *p* = 0.0005, I^2^ = 69%) were lower in the ACLR group compared to controls.

**Conclusions:**

After ACLR, the observed reduction in peak knee flexion angle suggests a walking pattern with a straighter knee, or a less erect gait posture, which can persist for ≥ 12 months post surgery. The lower sagittal plane knee moments suggest a less dynamic gait, characterized by reduced muscular demand. The small deviations in gait biomechanics from normal are lasting but inconsistent between studies. While associated with long-term outcomes like post-traumatic osteoarthritis, the direct clinical relevance of these specific gait changes requires further study.

**Supplementary Information:**

The online version contains supplementary material available at 10.1186/s40798-026-01016-x.

## Key Points

Injuries to the anterior cruciate ligament (ACL) are often treated through surgery.

This study revealed lower peak knee flexion angles and peak knee extension moments during the initial stance phase of walking in patients with ACL reconstruction compared to healthy controls

Some of these biomechanical gait changes remained up to 12 months post-surgery.

These findings confirm that gait alterations can still be detected over longer periods post ACL reconstruction. Future studies should focus on developing targeted rehabilitation programs to correct specific surgery-related deficits during walking. Moreover, longitudinal studies are needed to test whether such corrections mitigate adverse outcomes like post-traumatic knee osteoarthritis.

**Supplementary Information:**

The online version contains supplementary material available at 10.1186/s40798-026-01016-x.

## Introduction

Following a traumatic knee joint injury, anterior cruciate ligament reconstruction (ACLR) is a common medical treatment to restore knee joint stability and joint range of motion levels to pre-injury levels. Previously, it has been shown that ACLR has the potential to restore joint stability [1, 2], but does not attenuate the risk of sustaining knee osteoarthritis post-injury [3, 4]. Of note, between 30 and 80% of ACLR patients develop knee osteoarthritis within 10–15 years post-surgery [3–5]. Moreover, ACLR patients showed greater knee loading asymmetries while walking when compared with healthy controls [6]. During the first 12 months post-surgery, these asymmetries likely arise from a combination of factors, including ongoing pain, effusion, strength and range-of-motion deficits, and fear-avoidance behaviors, alongside early structural changes in the knee joint that may reflect the initial progression of osteoarthritis [6–9]. Subsequent rehabilitation of neuromuscular function to pre-injury levels can last up to a year but there is evidence that many athletes never return to their previous competitive level and ~ 25% of returners suffer a re-injury [10].

Given the inconsistent findings in the literature regarding biomechanical gait characteristics after ACLR, systematic reviews with meta-analyses were conducted to aggregate the available and somewhat controversial literature findings [11–14]. For instance in 2010, Shi et al. [11] examined the effects of ACLR on biomechanical gait characteristics in ACLR patients aged ≥ 18 years. Based on findings from 13 cross-sectional studies, the authors reported no significant differences in maximum knee flexion angle recorded during the loading phase between the ACLR and the healthy control group [11]. In 2017, Slater et al. [15] conducted a meta-analysis with the goal to examine three dimensional lower extremity kinematics and kinetics during walking in individuals with ACLR versus healthy controls by taking different time periods post-surgery into account (i.e., 3–64 months after ACLR). Twenty seven cross-sectional studies were included in the meta-analysis and the authors found lower peak knee-flexion angles together with lower external knee-flexion and knee-adduction moments during the early stance phase of walking, in the ACLR patients versus healthy peers [15]. The same group of authors additionally analyzed time periods post-surgery and observed lower peak knee-flexion angles, external knee-extensor and knee-adduction moments 9–42 months post ACLR [15]. Overall, these meta-analytical findings show altered biomechanical gait characteristics in the sagittal, frontal, and transverse planes, with differences persisting even 12 months post ACL surgery [13, 14].

However, the above described meta-analyses are methodologically limited in as much as the authors examined knee sagittal and transverse plane kinematics and moments during level walking only [11] or because they primarily included case control or cohort studies and assessed kinematic but not kinetic data [15]. Nine years have passed since the last meta-analysis on this topic [15] and during that time the available knowledge base has increased substantially. Accordingly, it is timely to re-analyze the scientific literature and provide an update on the current state of knowledge.

A better understanding of biomechanical gait characteristics differences between ACLR participants and healthy controls may help clinicians and researchers to design evidence-based and targeted rehabilitation programs. Here, we aimed to analyze kinematic and kinetic variables recorded during the stance phase of walking in male and female adults with ACLR versus healthy controls in the form of a systematic review with meta-analysis. As a primary hypothesis, we expected that ACLR individuals would demonstrate altered lower limb kinematics and kinetics during walking [11]. A secondary research question was to determine whether the identified biomechanical changes are transient (i.e., resolve during rehabilitation) or robust (i.e., persist over longer periods) by comparing outcomes across specific post-surgical time frames. We hypothesized reduced sagittal plane knee motion and joint moments even in the long-term (≥ 12 months) in ACLR individuals compared with healthy controls [15].

## Methods

The standard PRISMA (Preferred Reporting Items for Systematic Reviews and Meta-Analyses) guidelines were followed when conducting this systematic review with meta-analysis [16]. The protocol for this systematic review with meta-analysis was registered with PROSPERO on February, 1 st 2024 (Project: https://www.crd.york.ac.uk/prospero/display_record.php?RecordID=625699, ID: CRD42024625699). In this systematic review with meta-analysis, biomechanical gait patterns were assessed 0–<6 months, ≥ 6–12 months, and ≥ 12 months post-surgery.

### Eligibility Criteria

EndNote 20 software (Bld 14672, Clarivate, Philadelphia, PA, USA) was used for reference management, deduplication, and screening of potentially eligible papers. A PECOS (participants, exposure, comparators, outcomes, and study design) approach was applied to define inclusion and exclusion criteria (Table 1) a priori [17, 18]. Only studies including participants aged ≥ 18 years were included to minimize confounding effects related to skeletal immaturity and developmental gait adaptations. Observational cohort studies without healthy comparison groups were excluded because between-group comparisons are required to calculate standardized effect sizes for meta-analysis. Prospective cohort studies were excluded only when they lacked isolated cross-sectional biomechanical data comparing ACLR participants and healthy control group. Cohorts providing baseline or single-time-point gait data were eligible and included. To be eligible for inclusion in this meta-analysis, articles had to be published in peer-reviewed journals in English language.

**Table 1:** Adapted PECOS (participants, exposure, comparators, outcomes, and study design) framework for study inclusion and exclusion criteria

PECOS Framework	Inclusion	Exclusion
Participants	Male and female individuals aged ≥ 18 years who underwent anterior cruciate ligament reconstruction (ACLR) with any type of graft after isolated ACL injury or in addition to meniscus and/or collateral ligament injury	Individuals with adverse health events other than ACLR (e.g., injuries, recent surgery); individuals with neurological, systemic, or degenerative conditions; individuals aged < 18 years
Exposure	ACL surgery	–
Comparator	Between subject comparison with healthy controls (i.e., no ACL surgery)	Absence of a control condition
Outcome	Lower limb kinematics and kinetics (e.g., 3D motion capture and force plates, markerless motion capture, force plates only) during the walking stance phase	Measures of lower limb kinematics and kinetics during activities other than walking (e.g., running, jumping); measures of lower limb kinematics and kinetics during the swing phase of walking; no measures of lower limb kinematics and kinetics
Study design	Case control studies, case series, cross-sectional studies, randomized controlled trials (baseline), cohort studies.	Case studies, systematic reviews, cohort study, case series, randomized controlled trials

### Systematic Search

The following five databases were systematically searched from inception until January 2026: Scopus, PubMed, EMBASE, Physiotherapy Evidence Database (PEDro), and Cochrane Central Register of Controlled Trials (CENTRAL) (Appendix 1: Tables S1-S5). Grey literature sources (e.g., conference proceedings) from Google Scholar, Science Direct, Clinicaltrial.gov, PROQUEST and reference lists of already identified articles, were systematically screened for more articles to be eligible for inclusion. The literature search was developed for PubMed and adapted to each database (Appendix 1- supplementary material). The search syntax was created using the PECOS scheme and free-text keywords as well as medical subject headings (Mesh terms). Keywords and Mesh terms were combined using a Boolean search syntax and the operators AND, OR.

### Study Selection

Titles and abstracts were reviewed independently by two authors of this paper (A.E., and A.J.) to identify potentially eligible studies according to the a priori defined inclusion and exclusion criteria (Table 1). In cases where titles and abstracts did not provide sufficient information, full texts were examined. The same two authors then independently assessed the full texts for final inclusion. Discrepancies at any stage were resolved through discussion and consensus. When consensus could not be reached, a third reviewer (H.S) was involved to achieve a final decision.

### Quality Assessment

The methodological quality of the included studies was evaluated by the same two authors (A.E., A.J.) using a modified version of the Downs and Black checklist for non-randomized controlled trials [19]. The modified checklist includes 18 questions with eight reporting items (items 1, 2, 3, 4, 5, 6, 7, 10), two items for external validity (items 11 and 12), four items for internal validity (Bias) (items 15, 16, 18, 20), three items for internal validity-confounding (items 21, 22, 25), and one item for power (item 27). The items were scored as 0 (“no” and “unable to determine”), and 1 (“yes”), except for item 5 for the principal confounders which was scored 0 (“no”), 1 (“partially”), 2 (“yes”). The overall quality score of each study was calculated based on a percentage of the maximum score (19). In cases where there were discrepancies in the authors’ rating of the quality scores, consensus was reached through discussion. Studies with quality scores of 75% or higher were considered high quality, those with scores between 60% and 74% were classified as moderate quality, and those with scores of 60% or lower were categorized as low quality [20]. Studies were not excluded from meta-analysis based on quality. Interrater agreement for the quality assessment was evaluated using Cohen’s kappa (κ) statistic for each domain of the modified Downs and Black checklist. The level of agreement was quantified as slight (0.00 to 0.20), fair (0.21 to 0.40), moderate (0.41 to 0.60), substantial (0.61 to 0.80), and almost perfect (0.81 to 1.00) [21]. Disagreements were resolved with a predefined adjudication rule. Accordingly, a third senior reviewer (H.S.) was included to achieve a final decision. Quality scores were used for descriptive purposes and were not applied as analytic weights. The most frequently observed limitations involved incomplete confounder control (e.g., walking-speed prescription, graft type) and inadequate reporting of gait-acquisition protocols.

### Assessment of Publication Bias

Publication bias and potential asymmetry of the effect size were visually assessed using a contour-enhanced funnel plot after reviewing the meta-analytic data [22] (Appendix 3 - supplementary material). The relationships between effect sizes and standard error were statistically verified using Egger’s regression to determine whether the funnel plot was asymmetric [23]. In the case of asymmetry, the mean effect size obtained by adjusting the asymmetry was calculated using the trim-and-fill method and compared with the original average effect size to determine the number of missing studies [24].

### Data Collection

Two reviewers independently (A.E. and A.J.) extracted all relevant data (participants, control, number of participants, sex, graft, time since surgery, time since injury, walking protocol, and outcomes related to kinematic and kinetic data) from the included articles. For studies requiring plot digitization, two independent digitizations were performed and averaged. Inter-extractor reliability was excellent (ICC = 0.95). The current review extracted kinematics and kinetics only during the stance phase of gait. No data are reported during the swing phase. Kinematic variables included peak joint angles, and joint excursions in the stance phase gait. Kinetics comprised peak joint moments and ground reaction forces during the stance phase of gait. All kinetic data for joint moments were extracted as external joint moments. The sign convention was standardized so that an external moment tending to flex a joint is positive, and an external moment tending to extend a joint is negative. For studies that reported the absolute value of the peak extension moment, the sign was reinstated to maintain this consistent convention. The peak knee flexion moment refers to the maximum positive external flexion moment. The peak knee extension moment refers to the minimum (most negative) external moment, representing the peak external demand for knee extension. These represent two distinct temporal and biomechanical events and were therefore analyzed separately.

Kinetic variables related to joint moments included peak external joint moments only. Measures such as joint moment impulses or integrated loading parameters were not included because these outcomes were inconsistently reported across studies and would have increased methodological heterogeneity. Moreover, the other outcomes were not reported in at least five studies as indicated in the pre-registration trial. Peak values were defined as the maximum values observed across the entire stance phase rather than at discrete gait events such as heel strike. These decisions were made to maximize comparability across studies and minimize outcome definition heterogeneity. To ensure the independence of data points and avoid double-counting of participants from overlapping cohorts, the following steps were taken in accordance with the Cochrane guidelines [25]. If multiple publications by the same author group reported on an identical participant cohort (identified by matching sample size, demographic data, and recruitment period), only one dataset was included per outcome. The publication with the most comprehensive or directly relevant data for the outcome measure was selected. Studies that stratified a single cohort into mutually exclusive subgroups (e.g., by time post-surgery or graft type) were included with each subgroup analyzed separately, provided they were compared against a shared or equivalent control group.

In cases where authors from potentially eligible studies did not sufficiently report data, corresponding authors were contacted and asked to share the missing data. If the authors did not respond, they were reminded one more time and if they still did not respond, the study was excluded. Relevant data not reported in text or tables but in figures were extracted using a freeware and web-based plot digitizer [26]. For the sub-analyses according to time post-surgery, the included studies were categorized as short-term (0–<6 months), mid-term (≥ 6–12 months), and long-term (≥ 12 months). In several included studies, multiple ACLR subgroups according to graft type or sex were analyzed within the same investigation. When such subgroup data represented mutually exclusive participant samples, each subgroup was included separately in the meta-analysis to preserve data independence.

Data were meta-analyzed for the following kinetic and kinematic outcome measures assessed during the stance phase of walking: peak knee flexion angle, peak knee adduction angle, peak hip flexion angle, peak vertical ground reaction force, peak knee flexion moment, peak knee extension moment, peak knee adduction moment. In cases where fewer than five studies were identified for a specific outcome measure (e.g., ankle joint power), data were not meta-analyzed. Although meta-analysis can be performed with fewer studies, pooled estimates derived from very small study numbers may produce unstable variance estimates and unreliable heterogeneity measures [27].

### Statistical Analyses

Quantitative data synthesis was computed and illustrated in the form of forest plots using the Cochrane Review Manager 5.1 (The Nordic Cochrane Centre, The Cochrane Collaboration, Copenhagen, Denmark). To examine the main research question, between-group standardized mean differences (SMD) with 95% confidence intervals (CI) were computed as effect size measures using a random-effects model to elucidate the gait biomechanical differences between ACLR patients and healthy controls. In addition to SMD, the mean differences (ACLR mean minus healthy control mean) were used to report group differences in original units (e.g., degrees). Mean differences were calculated using a random-effects, inverse-variance model. To explore potential sources of heterogeneity, subgroup analyses regarding differences in biomechanical gait characteristics were analyzed short-term (0–<6 months), mid-term (≥ 6–12 months), and long-term (≥ 12 months). These subgroup analyses were conducted as sensitivity analyses to evaluate whether pooled effect sizes differed across recovery stages and were interpreted as exploratory assessments rather than causal temporal effects. The SMD were categorized as trivial (0–0.2), small (0.2–0.5), moderate (0.5–0.8), and large (> 0.8) [28–30]. Study heterogeneity was assessed using Cochran’s Q statistic (with degrees of freedom (df) and p-value), which tests for the presence of heterogeneity, and the I² index, which quantifies the proportion of total variability in the effect estimates that is due to heterogeneity rather than sampling error (chance). The between-study variance was estimated using τ². The level of heterogeneity based on I² was classified as high (> 75%), moderate (50%–75%), and low (25%–50%) [31].

## Results

### Study Selection

**Fig. 1:**
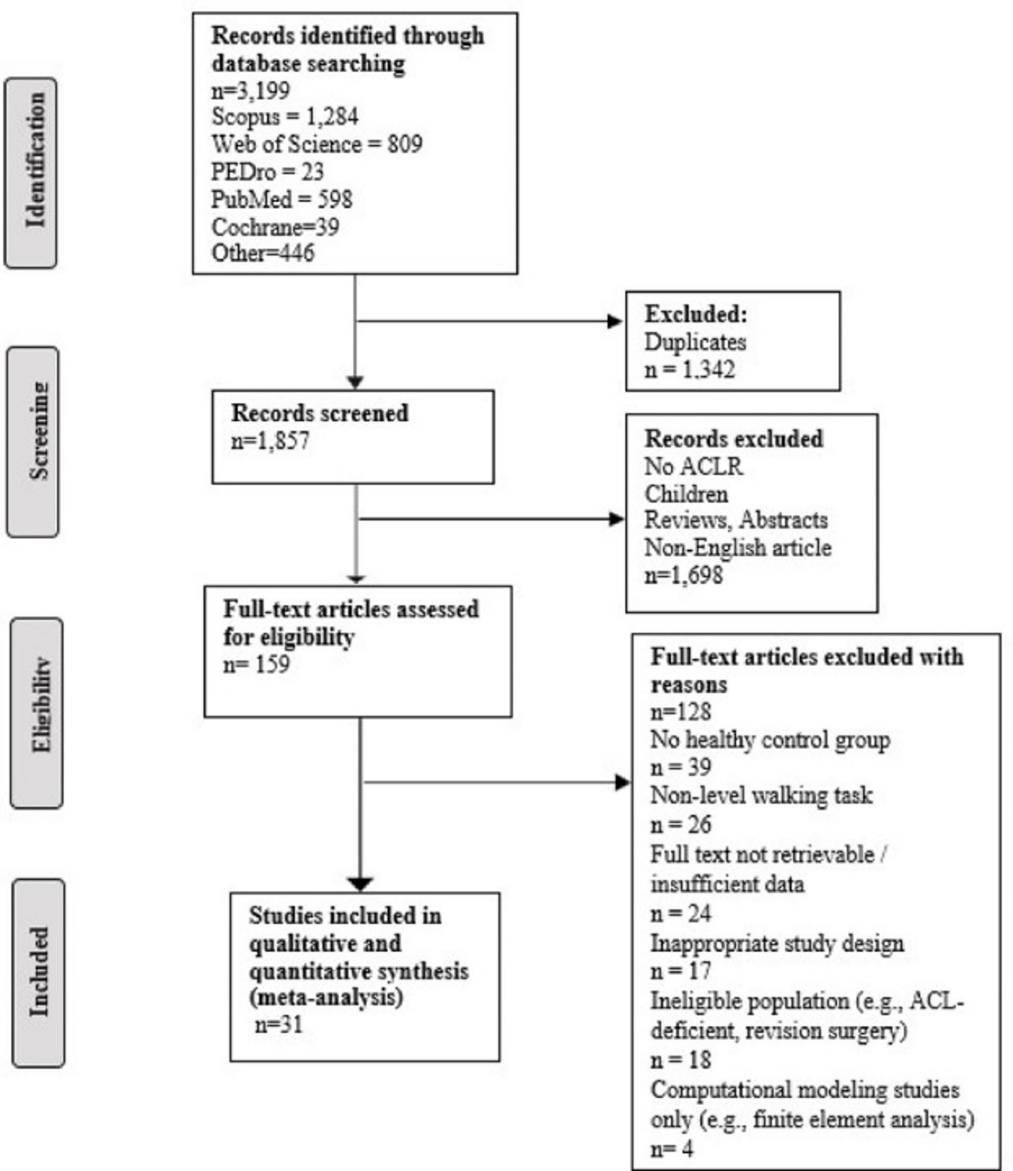
PRISMA flow chart illustrating the study selection process

The initial search identified 3199 studies. After duplicate removal, 1857 studies remained. Following the screening of titles and abstracts, 159 full texts were further considered. Finally, 31 cross-sectional studies with 1024 ACLR patients and 701 healthy controls were eligible to be included in this systematic review with meta-analysis (Fig. 1).

### Study Characteristics

Table 2 shows the characteristics of the included studies. Five studies were classified as short-term [32–36], eight studies as mid-term [37–44], and 19 studies as long-term [38, 45–62]. Overall, 31 studies reported kinematic data and 30 studies kinetic data.

Two studies did not provide information on the participants’ sex [39, 59], one study did not specify the type of graft used [48], and 18 studies did not report the time since injury [36–39, 44–46, 48–50, 52–59, 61, 62]. All studies used three-dimensional motion analysis (kinematics). Kinetic data were assessed in almost all except one study [44] using force plates integrated into the treadmill or positioned in the center of a walkway to evaluate ground reaction forces while walking. The three studies published by Ferber et al. report on the same participant cohort [32–34]. For each meta-analyzed outcome, data from only one of these studies were included to maintain sample independence. A dataset comprising three mutually exclusive subgroups, early, mid, and late post-ACLR, was analyzed separately using time-based subgroup analyses [52].

**Table 2:** Summary table of the included 31 cross-sectional studies used for quantitative biomechanical gait analyses of adult male and female patients with anterior cruciate ligament reconstruction versus healthy controls

Study	Number of ACLR Participants,	Sex (male/female), age, year (mean ± SD or range)	Number of healthy controls	Sex (male/female), age, year (Mean ± SD or range)	Graft type	Time sincesurgery	Gait protocol		Outcomes
Asaeda et al. [45]	Male: 16 and Female: 16	M: 29.1 ± 11.7 and F: 25.6 ± 13.0	16 males and 16 females	Male: 25.0 ± 6.8 and Female: 21.3 ± 1.8	QST	12 months	5 trials, 10-m walkway, self-selected speed	Tibia rotation angle, maximum knee flexion during the loading response, minimum knee flexion at mid-stance and excursion of knee motion	
Bulgheroni et al. [46]	15	15 M, 25 ± 3	5	5 M, 28 ± 3	BPB technique	17 ± 5.0 months	5 trials, 20-m walkway	Lower limb joint angle and moments	
Bush-Joseph et al. [47]	22,	13 M/9 F, 27 ± 11	22	13 M/9 F, 29 ± 8	Autogenous PT	22. ± 12.0 months	6 trials, walkway, self-selected speed, speed of 1.18 ± 0.16 m/s in the ACLR and 1.16 ± 0.16 m/s in the control	Knee joint sagittal plane angles and peak external moments	
Butler et al. [48]	17	13 F/4 M, 23.4 ± 5.7	17	13 F/4 M, 23.4 ± 5.7	NR	1 year	Walkway, intentional gait velocity, 1.44 ± 0.11 in the ACLR, 1.45 ± 0.12 m/s in the control	Peak adduction and adduction excursion and peak abduction moments at the knee and hip	
Davis-Wilson et al. [49]	196	73 M/123 F, 21 ± 4	106	32 M/74 F, 21 ± 3	139 BPB autograft; 48 ST autograft; 3 QT autograft; 6 allograft	29.6 ± 30.2 months	5 trials, self-selected speed	Peak vertical ground reaction force, peak knee flexion angle, knee flexion excursion	
Dewig et al. [50]	47	13 M/34 F, 20.8 ± 3.2	47	13 M/34 F, 21.2 ± 2.3	23 PT, 22 HT 1 QT, 1 allograft	4 ± 3 years	5 trials, 6 m walking, self-selected speed, 1.31 ± 0.18 in the ACLR, 1.28 ± 0.15 m/s in the control	Kinetics (peak knee extension moment, Knee abduction moment, vertical ground reaction force) and kinematic (peak knee flexion angle and knee flexion displacement)	
Erhart-Hledik et al. [51]	17	5 M/12 F, 29.6 ± 7.3	17	5 M/12 F, 35.4 ± 7.4	15 AG, 1 BPB allograft, and 1 BPB autograft	2.2 ± 0.3 years	3 trials, self-selected speed, 10-m walkway	Knee kinematics and kinetics	
Ferber et al. [32]	10	5 M/5 F, 27.7 ± 9.1	10	5 M/5 F 24.4 ± 3.1	BPB	3 months	12 trials, 5 m wooden walkway, Self-selected pace	Hip and knee joint moments, powers, and positions	
Ferber et al. [33]	10	5 M/5 F, 27.7 ± 9.1	10	5 M/5 F 24.4 ± 3.1	BPB	3 months	12 trials, 5 m wooden walkway, self-selected pace	Hip and knee joint moments, powers, and positions	
Ferber et al. [34]	10	5 M/5 F, 27.7 ± 9.1	10	5 M/5 F 24.4 ± 3.1	BPB	3 months	12 trials, 5 m wooden walkway, self-selected pace	Hip and knee joint moment, power, and position	
Gao et al. [37]	14	12 M/2 F, 25.1 ± 5.9	15	12 M/3 F, 22.8 ± 2.6	7 HT autografts, 5 BPB allografts, and 2 AG	Within 12 months	5 trials, self-selected speed	Peak knee flexion kinematics	
Georgoulis et al. [44]	21	19 M/2 F, 25 ± 4	10	8 M/2 F, 24.7 ± 3.7	BPB	30 ± 16.9 weeks	6 trials, self-selected pace on a 10-meter walkway	Knee flexion, tibial adduction-abduction, and rotation	
Goetschius et al. [52]	A: Early ACLR: 18, B: mid ACLR: 20, C: late ACLR: 18	early ACLR: 7 M/11 F, 21.7 ± 4.1; mid ACLR: 4 M/16 F, 20.5 ± 2.2; late ACLR:6 M/12 F, 26.7 ± 4.4	20	7 M/13 F, 22.4 ± 3.2	Early ACLR: 7 PT, 10 HT, and 1 cadaver; mid ACLR: 11 PT, 5 HT, and 4 cadaver; late ACLR: 10 PT, 5 HT, and 3 cadaver	Early ACLR: 1.4 ± 0.4 year; mid ACLR: 3.3 ± 0.6 years; late ACLR: 8.6 ± 2.8 years	10 strides for each limb, at standardized speeds of 1.34 m/s on treadmill	Knee and hip kinetics and kinematics in the sagittal and frontal planes and vertical ground reaction forces	
Hadizadeh et al. [35]	33	13 M/9 F, 23.6 ± 5.4	15	9 M/6 F, 21.5 ± 1.0	HT	4 and 5 weeks	7 trials, 13 m walkway, intentional walking speed	Sagittal plane knee angles, vertical ground reaction forces, knee extension moments	
Hall et al. [53]	15	7 M/8 F, 26.0 ± 6.0	17	7 M/10 F, 26.0 ± 4.0	41% HT, 41% PT, 12% combination of cadaver and HT, and 6% cadaver	6 years	6 trials,10 m walkway, self-selected speeds	Knee flexion angle, maximum internal ankle plantar flexion, knee extension, hip extension, hip abduction, and external knee varus moments	
Kumar et al. [38]	37	22 M/15 F, 30.8 ± 5.3	13	9 M/4 F, 29.4 ± 8.3	27 ST autograft, 8 tibialis posterior allograft, 1 PT allograft, 1 ST allograft	6 and 12 months	4 trials, walked over-ground at a pace of 1.35 m/s	Peak knee adduction angle, knee adduction moment impulse, peak knee adduction moment	
Lewek et al. [36]	Weak-ACLR: 10; strong-ACLR: 8	Weak-ACLR: 4 M/6 F, 25.0 ± 7.8; strong-ACLR: 5 M/3 F, 21.4 ± 6.0	10	8 M/2 F, 32.2 ± 6.6	Allograft or double loop ST-GT autograft	Weak-ACLR: 20.8 (9.4) weeks Strong-ACLR: 14.3 (2.4) weeks	6 trials, 30-foot walkway, free-speed	Knee flexion angle and moment	
Milandri et al. [54]	15	15 M, 28.6 ± 6.8	15	15 M, 28.6 ± 6.8	HT autograft	4 and 6 years	5 trials, walkway, self-selected speeds	Hip and knee flexion angles, external hip and knee flexion moments, external knee adduction moment, and knee varus	
Noehren et al. [55]	20	20 F, 25.0 ± 6.2	20	20 F, 26 ± 5.1	8 HT, 7 PT, and 5 autograft	5.2 ± 3.2 years	5 trials, speed of 1.5 m/s treadmill	Hip flexion and knee flexion angles, knee extensor moment, hip extensor moment	
Pamukoff et al. [56]	High BMI ACLR: 26, Low BMI ACLR: 26	High BMI ACLR: 14 M/12 F, 23.8 ± 4.2 Low BMI ACLR: 14 M/12 F, 21.5 ± 2.2	High BMI: 26, Low BMI: 26,	High BMI: 14 M/12 F, 22.6 ± 2.9 Low BMI: 14 M/12 F, 22.0 ± 3.0	PT: 22; HT: 15; allograft: 15	Low BMI ACLR: 61.7 ± 27.4 months; High BMI ACLR: 65.1 + 29.5 months;	5 trials, walkway, preferred walking speed	Vertical ground reaction forces, knee flexion angle and external moment, and knee adduction moment	
Patterson et al. [57]	17 lower limbs of 14	14 F, 20.8 ± 1.2	17	F, 23.7 ± 3.1	8 HT auto-grafts and 9 BPB autograft	3.5 ± 3.2 years	10 trials, walkway, self-selected speeds	Hip, knee, and ankle joint angular displacements in 3D	
Ripic et al. [61]	16	7 FT-Q: 4 M/3F, 28.6 ± 7.3; 9 PT-Q: 8 M/1F, 25.2 ± 4.3	11	6 M/5F, 23.4 ± 4.8	QT autograft	FT-Q: 23.5 ± 10.7 months, PT-Q: 24.4 ± 11.7 months	3 trials, self-selected speeds, 10-m walkway	Peak knee flexion angle, sagittal knee range of motion, peak internal knee extension moment, and peak internal knee flexion moment	
Sanford et al. [58]	10	4 M/6 F Age was not mentioned	12	6 M/6 F	BPB or 1 HT	7.4 ± 5.8 years	3 trials, 10 m walkway, self-selected pace	Knee flexion, adduction, and internal rotation moments	
Saxby et al. [62]	104	66 M/38 F, 29.7 ± 6.5	60	35 M/25 F, 27.5 ± 5.4	Single-bundle ST and GT autograft	2.5 ± 0.4 years	3 trials, 10 m walkway, self-selected pace	External knee moments, knee angles, ranges of motion	
Timoney et al. [39]	10	20 and 30 years. Sex was not mentioned.	10	20 and 30 years	PT	10 months (range, 9 to 12).	Walkway, self-selected speed	Knee flexion moment, external knee moment	
Varma et al. [59]	12	30.5 ± 8.7	12	24.8 ± 8.8	Single bundle HT autograft	4.5 (3.5) years	3 trials, 7 m walkway, self-selected pace	Peak knee flexion and adduction moments	
Wang et al. [40]	Traditional transtibial reconstruction (TT): 11, anteromedial portal technique (AMP): 10	(TT): 6 M/5 F, 28.8 ± 10.3; (AMP): 7 M/3 F, 30.3 ± 7.1	20	15 M/5 F, 26.3 ± 7.7	HT	TT: 10.1 ± 2.9 months; AMP: 9.5 ± 3.2 months	≥ 3 trials, walkway, normal daily walking speed	Knee joint moments and shear forces, knee flexion	
Webster et al. [43]	Patellar tendon: 17, hamstring tendon: 17	Patellar tendon: 16 M/1 F, 23.8 ± 5.0; hamstring tendon: 16 M/1 F, 26.8 ± 8.0	17	17, 16 M/1 F, 24.7 ± 5.0	17 PT and 17 HT	Patellar tendon: 11 months; hamstring tendon: 9.3 months	3 trials, comfortable-speed	Sagittal plane kinematics and kinetics of the lower limb	
Webster et al. [41]	Patellar tendon: 18, hamstring tendon: 18,	Patellar tendon: 17 M/1 F, 23.8 ± 6.0; hamstring tendon: 16 M/2 F, 26.6 ± 6.0	18	18, 16 M/2 F, 24.7 ± 5.0	18 PT and 18 HT	Patellar tendon: 10.9 (2); hamstring tendon: 9.0 (2.4)	6 trials, comfortable-speed, walkway	Knee joint angles	
Webster et al. [42]	Patellar tendon: 16, hamstring tendon: 16,	Patellar tendon: M, 23.8 ± 6.0; hamstring tendon: M, 27.5 ± 6.0	16	16, M, 25.0 ± 5.0	16 PT and 16 HT	Patellar tendon: 11.2 ± 2; hamstring tendon: 9.4 ± 3	3 trials, comfortable-speed, walkway	Knee adduction moment, knee varus angle and vertical ground reaction force	
Zabala et al. [60]	45	26 M/19 F, 29.5 ± 6.1	45	26 M/19 F, 30.2 ± 4.7	AG	26 months	3 trials, comfortable-speed, walkway	Knee ad/abduction moment, flexion/extension moment, and external/internal rotation moment	

*ACLR* Anterior cruciate ligament reconstruction, *ROM *Range of motion, 2D: 2-dimensional; *3D* 3-dimensional, *NR* Not reported, *SD* Standard deviation, *M* Male, *F* Female, *AG* Achilles allograft, *PT* Patellar tendon, *HT* Hamstring tendon, *BPB* Bone-patellar tendon-bone graft, *CG* cadaver, *QST* Quadrupled semitendinosus tendon, *GT* Gracilis tendon, *QT* Quadriceps tendon, *ST* Semitendinosus

### Methodological Quality Assessment

The mean methodological quality of all included studies was moderate with a mean score of 72% on the Downs and Black checklist [19] (Table 3). Among the 31 included studies, eight were rated high-quality [38, 48–50, 53, 56, 61, 62], only one low quality [39], and the remaining studies were rated as moderate quality. Methodological quality findings varied across assessment domains. Most studies demonstrated strong reporting quality, with clear descriptions of objectives, outcomes, participant characteristics, and main findings (Table 3). Internal validity related to measurement bias was generally acceptable, as most studies employed appropriate statistical analyses and validated biomechanical outcome measures. However, external validity was frequently limited due to insufficient reporting regarding participant representativeness and recruitment procedures. Similarly, several studies demonstrated limited control or adjustment for potential confounding factors, particularly biomechanical variables such as walking speed and graft characteristics (Table 3). Authors from six studies computed a priori power analyses to estimate the required sample sizes [48–50, 56, 61, 62].

**Table 3:** Downs and Black methodological quality assessment scores of the 31 included studies

Study	Reporting								External validity		Internal validity (bias)				Internal validity (confounding)			Power	Score (%)	Quality
	1	2	3	4	5	6	7	10	11	12	15	16	18	20	21	22	25	27		
Asaeda et al. [45]	1	1	1	1	2	1	1	1	0	0	0	1	1	1	1	0	1	0	74	MQ
Bulgheroni et al. [46]	1	1	1	1	1	1	0	0	1	0	0	1	1	1	1	0	1	0	63	MQ
Bush-Joseph et al. [47]	1	1	1	1	2	1	1	1	0	0	0	1	1	1	1	0	1	0	74	MQ
Butler et al. [48]	1	1	1	1	2	1	1	0	0	0	0	1	1	1	1	1	1	1	79	HQ
Davis-Wilson et al. [49]	1	1	1	1	2	1	1	1	0	0	0	1	1	1	1	0	1	1	79	HQ
Dewig et al. [50]	1	1	1	1	2	1	1	1	0	0	0	1	1	1	1	1	1	1	84	HQ
Erhart-Hledik et al. [51]	1	1	1	1	2	1	1	1	0	0	0	1	1	1	1	0	1	0	74	MQ
Ferber et al. [32]	1	1	1	1	1	1	1	0	0	0	0	1	1	1	1	0	1	0	63	MQ
Ferber et al. [33]	1	1	1	1	1	1	1	0	0	0	0	1	1	1	1	0	1	0	63	MQ
Ferber et al. [34]	1	1	1	1	1	1	1	0	0	0	0	1	1	1	1	0	1	0	63	MQ
Gao et al. [37]	1	1	1	1	1	1	1	1	0	0	0	1	1	1	1	1	1	0	74	MQ
Georgoulis et al. [44]	1	1	1	1	1	1	1	1	0	0	0	1	1	1	1	0	1	0	68	MQ
Goetschius et al. [52]	1	1	1	1	1	1	1	0	0	0	0	1	1	1	1	0	1	0	63	MQ
Hadizadeh et al. [35]	1	1	1	1	1	1	1	1	0	0	0	1	1	1	1	0	1	0	68	MQ
Hall et al. [53]	1	1	1	1	2	1	1	1	1	0	0	1	1	1	1	0	1	0	79	HQ
Kumar et al. [38]	1	1	1	1	2	1	1	1	1	0	0	1	1	1	1	0	1	0	79	HQ
Lewek et al., [36]	1	1	1	1	1	1	1	1	0	0	0	1	1	1	1	0	1	0	68	MQ
Milandri et al. [54]	1	1	1	1	2	1	1	1	0	0	0	1	1	1	1	0	1	0	74	MQ
Noehren et al. [55]	1	1	1	1	2	1	1	1	0	0	0	1	1	1	1	0	1	0	74	MQ
Pamukoff et al., [56]	1	1	1	1	2	1	1	1	0	0	0	1	1	1	1	0	1	1	79	HQ
Patterson et al. [57]	1	1	1	1	2	1	1	1	0	0	0	1	1	1	1	0	1	0	74	MQ
Ripic et al. [61]	1	1	1	1	2	1	1	1	1	0	0	1	1	1	1	1	1	1	89	HQ
Sanford et al. [58]	1	1	1	1	2	1	1	1	0	0	0	1	1	1	1	0	1	0	74	MQ
Saxby et al. [62]	1	1	1	1	2	1	1	1	1	0	0	1	1	1	1	0	1	1	84	HQ
Timoney et al. [39]	0	1	1	1	1	1	1	0	0	0	0	1	1	1	1	0	1	0	58	LQ
Varma et al. [59]	1	1	1	1	2	1	1	1	0	0	0	1	1	1	1	0	1	0	74	MQ
Wang et al. [40]	1	1	1	1	2	1	1	1	0	0	0	1	1	1	1	0	1	0	74	MQ
Webster et al. [43]	1	1	1	1	2	1	1	1	0	0	0	1	1	1	1	0	1	0	74	MQ
Webster et al. [41]	1	1	1	1	1	1	1	1	0	0	0	1	1	1	1	0	1	0	68	MQ
Webster et al. [42]	1	1	1	1	1	1	1	1	0	0	0	1	1	1	1	0	1	0	68	MQ
Zabala et al. [60]	1	1	1	1	1	1	1	1	0	0	0	1	1	1	1	0	1	0	68	MQ
Average score (mean)																			72	MQ

1 = Yes; 0 = No; *SD* Standard Deviation, *HQ* High Quality (score ≥ 75%), *MQ* Moderate Quality (60% ≤ score < 75%), *LQ* Low Quality (score < 60%)

### Lower Limb Joint Kinematics Differences Between Anterior Cruciate Ligament Reconstructed and Healthy Control Group

#### Knee Joint Angles

**Fig. 2:**
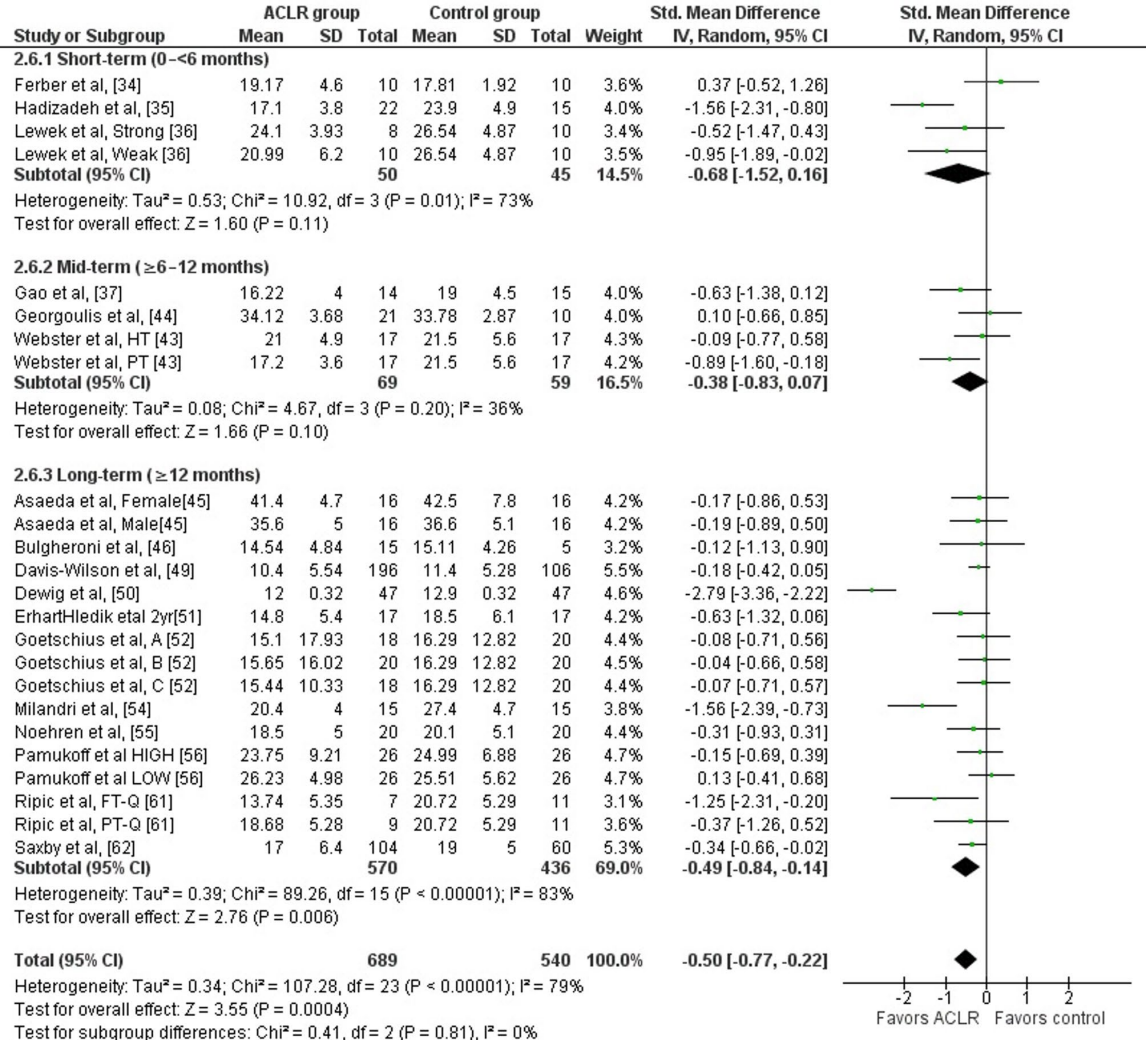
Forest plot illustrating the peak knee flexion angle difference during the stance phase of walking at preferred and constant speed in the ACLR versus healthy control group. Overall effects and the effects for the sub-analyses according to time post-surgery were calculated for each parameter as standardized mean difference (95% CI). Favors ACLR: lower in ACLR group; Favors control: lower in healthy control group; Weak: weak-quadriceps; Strong: strong quadriceps; HT: hamstring tendon graft; PT: patellar tendon graft; A: early ACLR; B: mid ACLR; C: late ACLR; HIGH: high body mass index; LOW: low body mass index; PT-Q: partial-thickness graft harvests; FT-Q: full-thickness graft harvests; SD: Standard deviation; Std: Standardized; CI: Confidence interval

Overall, lower peak knee flexion angles during the stance phase of walking were noted in ACLR patients compared to healthy controls (17 studies: small SMD=−0.50, 95% CI −0.77 to −0.22, *p* = 0.0004) [34–37, 43–46, 49–52, 54–56, 61, 62]. The analysis revealed high heterogeneity (Tau^2^= 0.34, Chi^2^= 107.28, df = 23, *p* < 0.0001, I^2^ = 79%). More specifically, the peak knee flexion angle was 1.99° (95% CI −2.84 to −1.13) lower in the ACLR compared to the control group (Fig. 2). Based on the results of the Egger’s test, there was no indication of publication bias (*p* = 0.53).

Even ≥ 12 months post-surgery, significantly lower peak knee flexion angles were observed in the ACLR patients versus healthy controls (11 studies: small SMD=−0.49, 95% CI −0.84 to −0.14, *p* = 0.006, I^2^ = 83%) [45, 46, 49–52, 54–56, 61, 62].

**Fig. 3:**
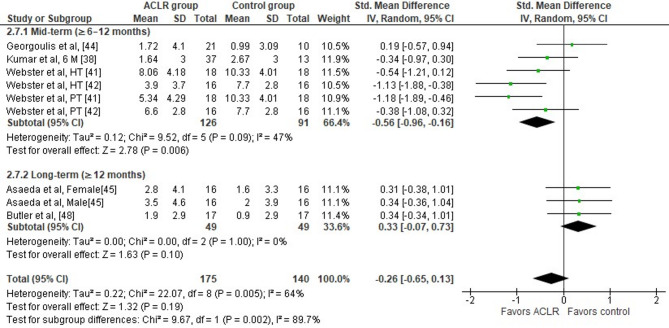
Forest plot illustrating the peak knee adduction angle difference during the stance phase of walking at preferred and constant speed in ACLR patients versus healthy controls. Overall effects and the effects for the sub-analyses according to time post-surgery were calculated for each parameter as standardized mean difference (95% CI). Favors ACLR: lower in ACLR group; Favors control: lower in healthy control group; HT: hamstring tendon graft; PT: patellar tendon graft; SD: Standard deviation; Std: Standardized; CI: Confidence interval

Researchers from six studies evaluated peak knee adduction angle differences during the stance phase of walking in ACLR patients versus healthy controls [38, 41, 42, 44, 45, 48]. Overall, the findings indicated no significant between group differences (six studies: small SMD=−0.26, 95% CI −0.65 to 0.13, *p* = 0.19) (Fig. 3). The analysis revealed moderate heterogeneity (Tau^2^= 0.22, Chi^2^= 22.07, df = 8, *p* = 0.005, I^2^ = 64%). Based on the results of Egger’s test, there was no indication of publication bias (*p* = 0.68).

Subgroup analyses showed that six to twelve months post-surgery, lower peak knee adduction angles (four studies: moderate SMD=−0.56, 95% CI −0.96 to −0.16, *p* = 0.006, I^2^ = 47%) were observed in ACLR patients versus healthy controls [38, 41, 42, 44]. More specifically, the peak knee adduction angle was 2.01° (95% CI −3.54 to −0.48) lower in the ACLR patients compared to the healthy control group up until six months post-surgery.

#### Hip Joint Angles

**Fig. 4:**
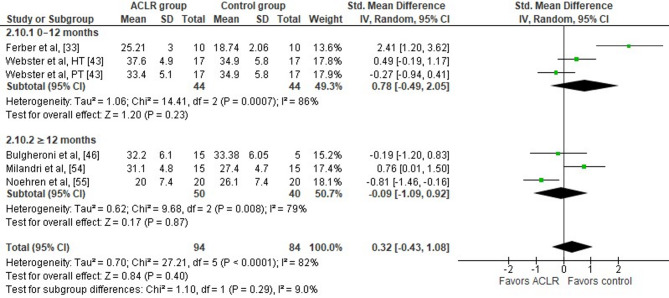
Forest plot illustrating the difference between peak hip flexion angle during the stance phase of walking at preferred and constant speed in the ACLR and the healthy control group. Overall effects and the effects for the sub-analyses according to time post-surgery were calculated for each parameter as standardized mean difference (95% CI). Favors ACLR: lower in ACLR group; Favors control: lower in healthy control group; HT: hamstring tendon graft; PT: patellar tendon graft; SD: Standard deviation; Std: Standardized; CI: Confidence interval

Overall, five studies evaluated peak hip flexion angle differences during the stance phase of walking in the ACLR and the healthy control group [33, 43, 46, 54, 55]. Overall, the findings indicate no significant between group differences (five studies: small SMD = 0.32, 95% CI −0.43 to 1.08, *p* = 0.40) (Fig. 4). The analysis further revealed a high level of study heterogeneity (Tau^2^= 0.70, Chi^2^= 27.21, df = 5, *p* < 0.001, I^2^ = 82%). Although, Egger’s regression test suggested funnel plot asymmetry (*p* = 0.01), trim-and-fill analysis did not impute any missing studies. This indicates that while small-study effects may be present, there is no statistical evidence from the trim-and-fill method that publication bias has substantively influenced the pooled estimate. However, given the inherent limitations of these tests, this finding should be interpreted cautiously [63].

### Lower Limb Joint Kinetic Differences Between Anterior Cruciate Ligament Reconstructed and Healthy Control Group

#### Normalized Vertical Ground Reaction Forces

**Fig. 5:**
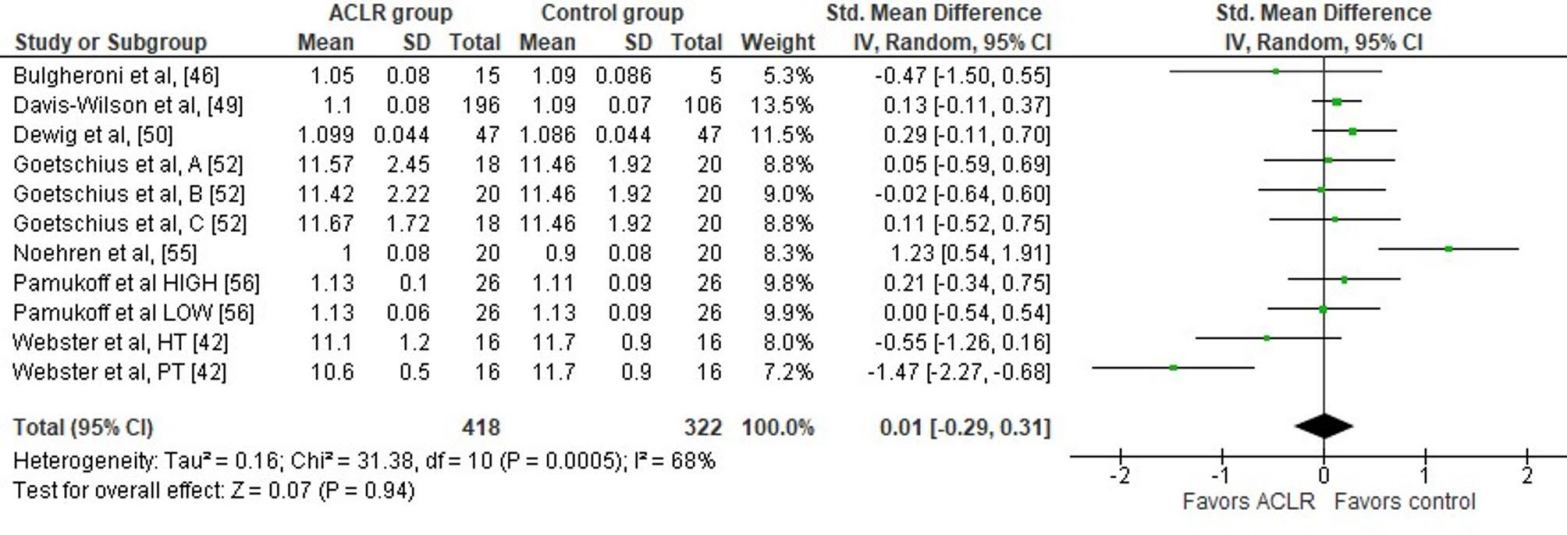
Forest plot illustrating the peak vertical ground reaction force difference during the stance phase of walking at preferred and constant speed in the ACLR and the healthy control group. The figure presents the overall difference and the difference for the sub-analyses according to time point post-surgery for each parameter as standardized mean difference (95% CI). Favors ACLR: lower in ACLR group; Favors control: lower in healthy control group; HT: hamstring tendon graft; PT: patellar tendon graft; A: early ACLR; B: mid ACLR; C: late ACLR; HIGH: high body mass index; LOW: low body mass index; SD: standard deviation; Std: standardized; CI: confidence interval

Seven studies evaluated peak vertical ground reaction force differences during the stance phase of walking between the ACLR and the healthy control group [42, 46, 49, 50, 52, 55, 56]. Findings indicated no significant between group differences (seven studies: trivial SMD = 0.01, 95% CI −0.29 to 0.31, *p* = 0.94) (Fig. 5). The analysis revealed moderate heterogeneity (Tau^2^= 0.16, Chi^2^= 31.38, df = 10, *p* = 0.0005, I^2^ = 68%). Based on the results of the Egger’s test, there is no significant evidence for publication bias (*p* = 0.20).

#### Knee Joint Moments

**Fig. 6:**
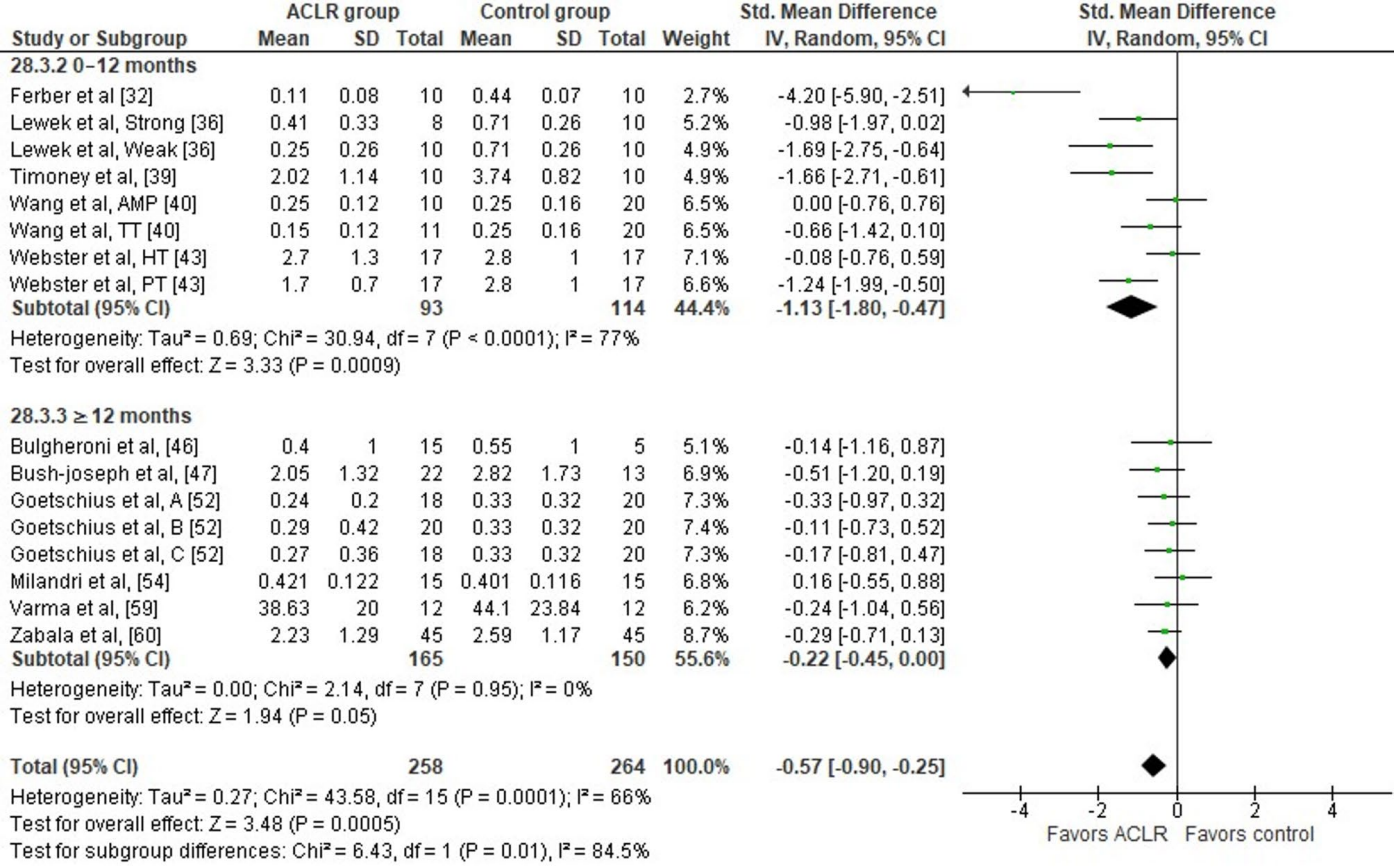
Forest plot illustrating the peak knee flexion moment difference during the stance phase of walking at preferred and constant speed between the ACLR and the healthy control group. The figure presents the overall difference and the difference for the sub-analyses according to time point post-surgery for each parameter as standardized mean difference (95% CI). Favors ACLR: lower in ACLR group; Favors control: lower in healthy control group; AMP: anteromedial portal technique; TT: traditional transtibial reconstruction; Weak: weak-quadriceps; Strong: strong quadriceps; HT: hamstring tendon graft; PT: patellar tendon graft; A: early ACLR; B: mid ACLR; C: late ACLR; SD: standard deviation; Std: standardized; CI: confidence interval

Eleven studies evaluated peak knee flexion moment differences between the ACLR and the healthy control group during walking [32, 36, 39, 40, 43, 46, 47, 52, 54, 59, 60]. The results showed significant moderate between group differences (11 studies: moderate SMD=−0.57, 95% CI −0.90 to −0.25, *p* = 0.0005) (Fig. 6). The analysis revealed moderate heterogeneity (Tau^2^= 0.27, Chi^2^= 43.58, df = 15, *p* = 0.0001, I^2^ = 66%). Overall, the peak knee flexion moment was lower in the ACLR compared to the healthy control group. Based on the results of the Egger’s test, there is significant evidence of publication bias (*p* < 0.001). The trim-and-fill method did not detect any missing studies, suggesting that publication bias is unlikely to have affected the meta-analytical results. This indicates that while small-study effects may be present, there is no statistical evidence from the trim-and-fill method that publication bias has substantively influenced the pooled estimate. However, given the inherent limitations of these tests, this finding should be interpreted cautiously [63].

The analysis of time post-surgery revealed that periods < 12 months (five studies: large SMD=−1.13, 95% CI −1.80 to −0.47, *p* = 0.0009, I^2^ = 77%) [32, 36, 39, 40, 43] post-surgery showed significantly lower peak knee flexion moment in the ACLR compared to the healthy control group.

**Fig. 7:**
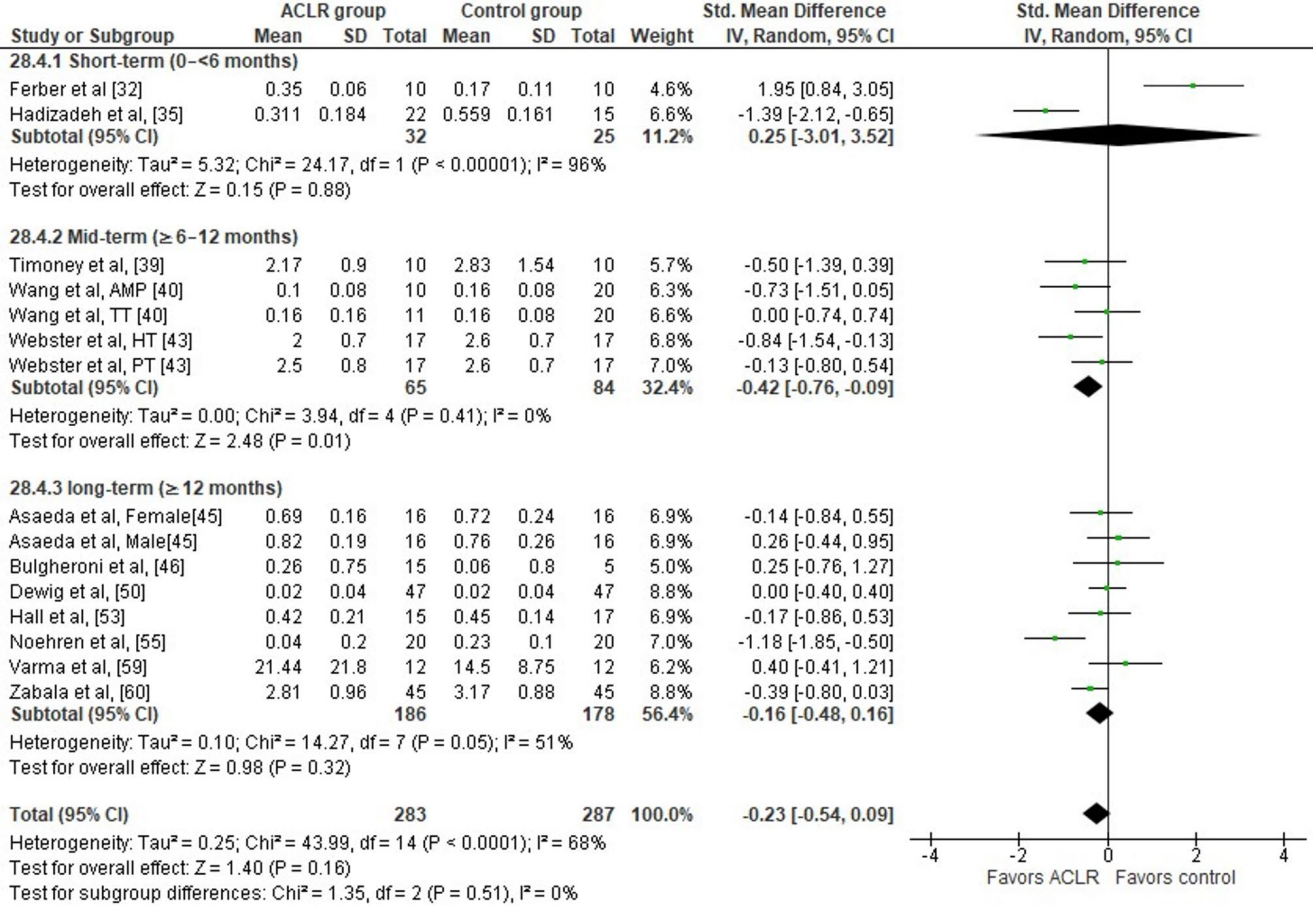
Forest plot illustrating the peak knee extension moment difference during the stance phase of walking at preferred and constant speed between the ACLR and the healthy control group. The figure presents the overall difference and the difference for the sub-analyses according to time point post-surgery for each parameter as standardized mean difference (95% CI). Favors ACLR: lower in ACLR group; Favors control: lower in healthy control group; AMP anteromedial portal technique; TT traditional transtibial reconstruction; HT hamstring tendon graft; PT patellar tendon graft; SD: standard deviation; Std: standardized; CI: confidence interval

Researchers from 12 studies evaluated peak knee extension moment differences during walking at preferred and constant speed in the ACLR and the control group [32, 35, 40, 43, 45, 46, 50, 53, 55, 59, 60]. Study findings indicated no significant between group differences (12 studies: small SMD=−0.23, 95% CI −0.54 to 0.09, *p* = 0.16) (Fig. 7). The analysis revealed moderate heterogeneity (Tau^2^= 0.25, Chi^2^= 43.99, df = 14, *p* < 0.0001, I^2^ = 68%). Based on the results of the Egger’s test, there is no evidence for publication bias (*p* = 0.15). In the time period 6–12 months post-surgery, significantly lower peak knee extension moment were observed in ACLR patients versus healthy controls (three studies: small SMD=−0.42, 95% CI −0.76 to −0.09, *p* = 0.01, I^2^ = 0%) [39, 40, 43].

**Fig. 8:**
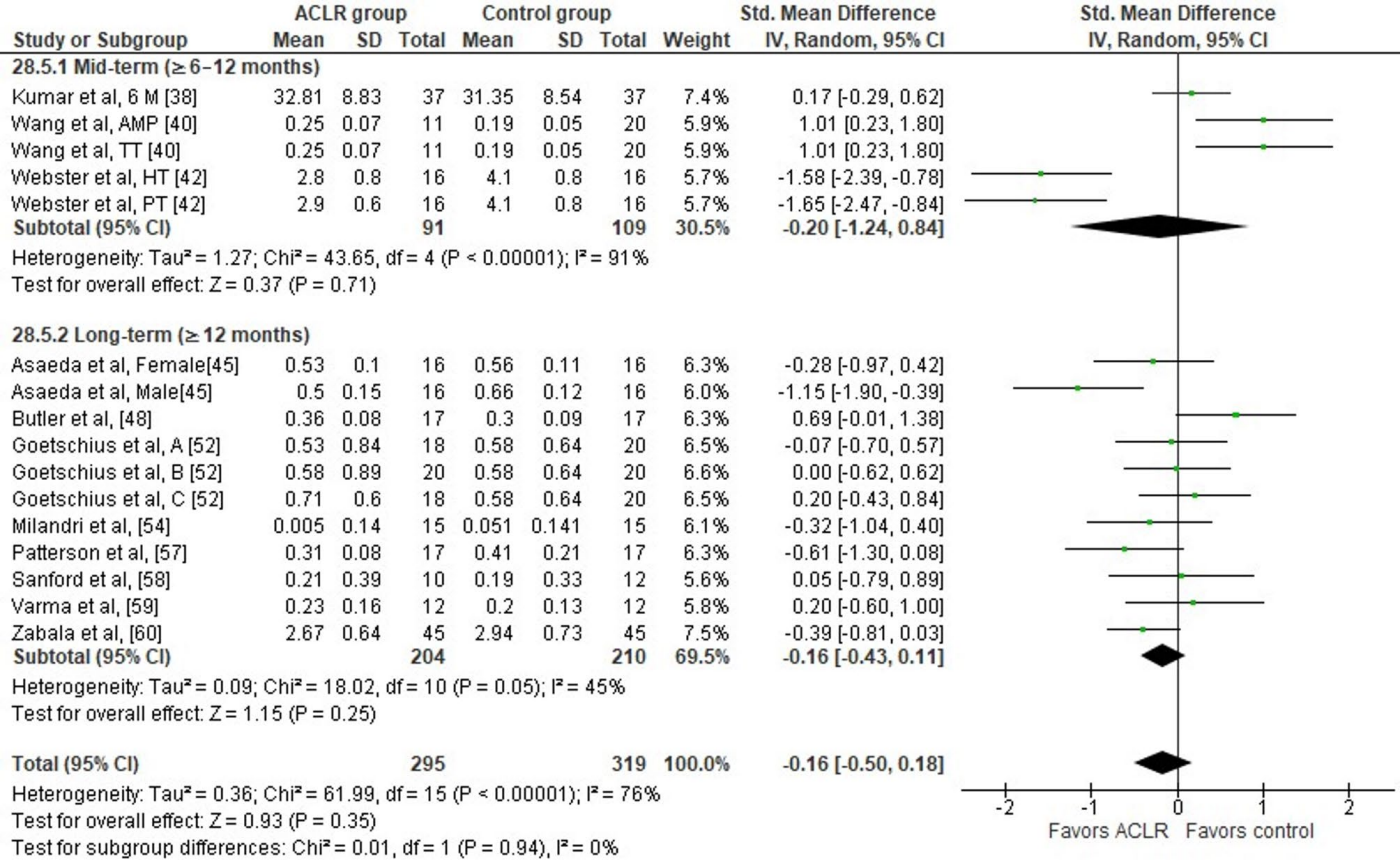
Forest plot illustrating the peak knee adduction moment difference during the stance phase of walking at preferred and constant speed between the ACLR and the healthy control group. The figure presents the overall difference and the difference for the sub-analyses according to time point post-surgery for each parameter as standardized mean difference (95% CI). Favors ACLR: lower in ACLR group; Favors control: lower in healthy control group; 6 M: 6 months post-surgery; AMP: anteromedial portal technique; TT traditional transtibial reconstruction; HT hamstring tendon graft; PT: patellar tendon graft; A early ACLR; B mid ACLR; C late ACLR; SD standard deviation; Std standardized; CI confidence interval

Eleven studies assessed peak knee adduction moment differences between the ACLR and the control group during the stance phase of walking [38, 40, 42, 45, 48, 52, 54, 57–60]. Total and subgroup analyses indicated no significant between group differences (11 studies: trivial SMD=−0.16, 95% CI −0.50 to 0.18, *p* = 0.25, I^2^ = 76%) (Fig. 8). The analysis revealed a high level of study heterogeneity (Tau^2^= 0.36, Chi^2^= 61.99, df = 15, *p* < 0.0001, I^2^ = 76%). Based on the results of the Egger’s test, there is no indication for publication bias (*p* = 0.68).

## Discussion

This systematic review with meta-analysis aimed to compare kinematic (e.g., peak knee flexion motion) and kinetic (e.g., peak knee moment and ground reaction forces) variables of the operated knee that received ACLR compared with healthy controls by taking short-, mid-, and long-term time periods post-surgery into account. With respect to the stance phase of gait, the main findings indicate (i) lower (*p* < 0.05) peak knee flexion angles in ACLR patients compared to healthy controls were not different early and mid-stage (*p* > 0.05) but were different (*p* < 0.05) beyond 12 months post-surgery, (ii) up until six months post-surgery, ACLR patients vs. healthy controls had lower (*p* < 0.05) peak knee adduction angles, (iii) regarding kinetic variables and irrespective of the time period, the peak joint moments for knee flexion were lower (*p* < 0.05) in the ACLR group compared to healthy controls, and (iv) at 6–12 months post-surgery ACLR patients vs. healthy controls had lower (*p* < 0.05) peak knee extension moment.

Overall, the current meta-analytical findings indicate significant differences for kinematic and kinetic variables between ACLR patients and healthy controls in the stance phase of gait. A secondary finding was that altered gait characteristics (i.e., peak knee flexion angle) are present past 12 months post-surgery. Importantly, the present findings indicate that sagittal plane knee underloading does not appear to restore completely even beyond 12 months post-ACLR, which may have important implications for long-term joint health and risk for knee osteoarthritis.

Persistent alterations in walking biomechanics following ACLR may have clinically meaningful implications, even when functional recovery appears satisfactory. Longitudinal evidence suggests that early gait asymmetries are associated with poorer long-term patient-reported outcomes. For example, asymmetrical knee kinematics and kinetics approximately two years post-ACLR have been linked to impaired knee symptoms and quality of life at extended follow-up [9]. Similarly, reduced loading symmetry during walking at six months post-surgery has been associated with inferior patient-reported outcomes at one year [6]. Emerging evidence also suggests that altered joint loading may influence structural joint health. Persistent underloading of the patellofemoral joint has been associated with adverse cartilage composition changes up to three years following ACLR [7], while insufficient knee loading during early recovery has been associated with unfavorable tibiofemoral cartilage profiles [8]. Clinically, these findings highlight the potential importance of monitoring gait biomechanics during rehabilitation to identify persistent loading asymmetries. However, as the current evidence is primarily observational, longitudinal and interventional studies are required to determine whether modifying these biomechanical alterations improves long-term clinical and structural outcomes.

### Differences Between Anterior Cruciate Ligament Reconstructed Individuals and Healthy Controls in Lower Extremity Joint Kinematics

This study revealed that the peak knee flexion angle is lower in ACLR patients versus healthy controls indicating that ACLR individuals change their knee biomechanics during walking which could be a consequence of insufficient neuromuscular knee joint control [64]. The lower peak knee flexion after ACLR patients is consistent with previous systematic reviews [11–13]. The reduced peak knee flexion angle observed during the stance phase in individuals following ACLR is consistent with previous systematic reviews and meta-analyses [11–14]. Persistent reductions in sagittal plane knee excursion likely reflect multifactorial adaptations rather than a single neuromuscular deficit. One important contributor may be persistent quadriceps dysfunction and orthogenic muscle inhibition. Such adaptations commonly occur following an ACL injury and reconstruction and can limit the ability to eccentrically control knee flexion during weight acceptance [13, 65]. Additionally, reduced knee flexion during the stance phase may represent a protective quadriceps avoidance strategy aimed at minimizing knee extensor loading and perceived joint instability [66]. Such adaptations are frequently accompanied by stiffened gait patterns characterized by reduced sagittal plane motion [67]. Behavioral and psychological factors may also contribute to persistent gait asymmetries. Fear of reinjury, reduced movement confidence, and avoidance behaviors have been shown to influence motor control strategies following ACLR and may contribute to long-term alterations in lower limb biomechanics [68]. Furthermore, biological factors such as graft characteristics, persistent joint symptoms, and low-grade inflammation or joint effusion may further disrupt neuromuscular function and movement coordination [69].

An external knee flexion moment causes the knee to flex. At the same time, knee extensor muscles become activated and produce an internal moment to resist the external flexion moment [12]. It is plausible that lower knee flexion angles evident in individuals post-ACLR may be related to altered quadriceps and/or hamstring muscle activation patterns in this patient population [12]. However, this conclusion is speculative, since this systematic review did not examine muscle activation patterns. The broad categorization of long-term follow-up (> 12 months) may have grouped participants at substantially different recovery stages. For example, a previous study reported outcomes at approximately 1, 3, and 8 years post-ACLR, indicating that biomechanical adaptations may evolve over even long periods [27]. The peak knee flexion angle was 1.81° lower in the ACLR compared to the control group. However, the observed difference of 1.99° is below the clinically meaningfulness difference of 3° [54, 70]. Importantly, reductions in peak knee flexion angle may not occur in isolation but often coincide with reductions in knee flexor moments, further supporting the presence of a coordinated sagittal plane unloading following ACLR.

Furthermore, with regards to the time period up until six months post-surgery, the peak knee adduction angle was 2.01° lower in the ACLR patients compared to the healthy control group. The observed gait adaptations may represent protective neuromuscular strategies aimed at modifying joint loading patterns. Such adaptations may reduce peak loading but could also result in altered load distribution across joint structures, potentially influencing long-term joint health [41]. On the other hand, greater knee abduction angle (equivalent to lower knee adduction angle) increases the risk of non-traumatic lateral knee osteoarthritis progression [71]. However, this finding should be interpreted with caution, since it unknown whether the cohorts who were included in this systematic review, had valgus malalignment pre-surgery. A previous meta-analysis indicated no significant between-group differences (ACLR individuals vs. healthy controls) for knee adduction angle [72]. Another systematic review and meta-analysis revealed strong evidence of lower peak knee adduction angles (i.e., less knee varus) in individuals 6–12 months post-ACLR with hamstring-tendon graft compared to healthy controls, but no evidence in those with a patellar-tendon graft [12]. An explanation for this combination of variables would be that the ACLR group walked with a more anteriorly-tilted pelvis [54]. The peak knee flexion angle (at all time periods post-surgery), peak knee adduction angle (up until six months post-surgery) could be identified as potentially modifiable gait characteristics in ACLR knees. Identifying potentially modifiable gait characteristics in ACLR patients may facilitate the development of specific interventions to prevent early onset and progression of post-traumatic knee osteoarthritis or re-injuries.

### Differences Between Anterior Cruciate Ligament Reconstructed Individuals and Healthy Controls in Lower Extremity Joint Kinetics

This meta-analysis identified lower peak knee flexion moments in the ACLR compared with healthy controls. When interpreted alongside the reduced peak knee flexion angles identified in the present review, these findings suggest that ACLR individuals adopt a stiffened sagittal plane movement characterized by simultaneous reductions in joint excursion and joint loading. Such coupled reductions in kinematics and kinetics are frequently observed during early and even long after ACLR may represent protective neuromuscular strategies designed to reduce quadriceps demand and perceived joint instability. However, sustained reductions in sagittal plane loading may alter cartilage mechanobiology and contribute to progressive joint degeneration.

Neurophysiological changes following ACL injury and reconstruction have been shown to alter lower limb muscle activation [73], accompanied by lower cortical activation in the injured limb [74]. The presence of pain, reported even at 2–5 years after surgery [75], or reflex inhibition [76], could further influence the movement patterns, as apparent with reduced flexion moments. Furthermore, compensatory movements at the hip may also decrease knee joint loading [77]. It is thus likely that multiple mechanisms contribute to long-term decreased flexion moments in the ACLR knees. It seems that quadriceps weakness is associated with altered knee joint moments [36, 47, 78]. Moreover, at 6–12 months post-surgery, significantly lower peak knee extension moments were observed in ACLR patients versus healthy controls. Lower peak knee extension moment in ACLR patients may be related to peripheral changes in the muscle–tendon units of the quadriceps muscle [79]. These peripheral changes may include chronic atrophy, changes in the compliance of the series elastic components of the muscle–tendon units, and alterations in the architectural structure and composition (fiber type) of the quadriceps muscle [79]. Moreover, quadriceps activation failure has been implicated as a source of lingering quadriceps weakness after ACLR [80, 81]. The present meta-analyses indicated no significant between group differences in peak vertical ground reaction force and peak knee adduction moment at both total and subgroup analyses. These results are consistent with Hart et al. [12], who recently reported that ACLR may not affect frontal plane moments. High knee adduction moment is an important risk factor for progression of non-traumatic knee osteoarthritis [82, 83]. However, osteoarthritis following ACLR appears to involve multifactorial and compartment-specific biomechanical alterations. Increasing evidence highlights the patellofemoral joint as a commonly affected site following ACLR. Reduced sagittal plane knee motion and joint loading may influence patellofemoral contact forces and cartilage health, potentially contributing to long-term symptoms and structural joint changes [84]. Accordingly, evaluation of osteoarthritis risk following ACLR should consider both frontal and sagittal plane loading patterns. The absence of significant between-group differences in knee adduction moment observed in the present meta-analysis suggests that frontal plane loading alterations may not consistently characterize gait biomechanics following ACLR. This finding may reflect the compartment-specific prevalence of post-traumatic knee osteoarthritis, with a higher prevalence of lateral tibiofemoral and patellofemoral osteoarthritis reported following ACL injury compared with non-traumatic osteoarthritis populations [85–87]. Importantly, these findings do not imply that frontal plane joint moments lack clinical relevance, but rather indicate that sagittal plane underloading strategies may represent an additional and potentially important biomechanical adaptation following ACLR. Therefore, rehabilitation strategies may benefit from targeting altered sagittal plane knee joint moments while continuing to monitor frontal plane mechanics. Nevertheless, longitudinal and interventional studies are required to deduce appropriate clinical implications.

### Sagittal Plane Knee Underloading Following Anterior Cruciate Ligament Reconstruction

The kinematic and kinetic data collectively indicate a consistent pattern of sagittal plane knee underloading following ACLR. Reduced peak knee flexion angles frequently co-occurred with reduced knee flexor moments, suggesting a coordinated movement strategy characterized by a stiffer gait pattern and reduced muscular demand during stance phase. This co-occurring kinematic-kinetic adaptation is consistent with emerging evidence demonstrating that reduced sagittal plane loading following ACLR may influence cartilage health and contribute to the development of post-traumatic osteoarthritis.

Longitudinal investigations have shown that lower patellofemoral contact forces, smaller knee flexion angles, and reduced knee flexion moments during walking early after ACLR are associated with worse cartilage composition at later follow-up periods [69]. Similarly, reduced joint loading following ACL injury and reconstruction has been associated with early structural joint degeneration and may represent a critical modifiable factor influencing osteoarthritis development [88]. Furthermore, prospective data indicate that individuals who later develop radiographic osteoarthritis demonstrate lower knee joint loading and reduced compartmental contact forces during early recovery following ACLR [66].

Evidence also suggests that abnormal loading patterns may relate to persistent knee symptoms following ACLR. The authors of a previous study reported that individuals with clinically relevant knee symptoms exhibit altered vertical ground reaction force patterns during walking, supporting the concept that both excessive and insufficient mechanical loading may negatively influence long-term joint health [65]. Collectively, these findings reinforce the potential clinical importance of identifying and addressing persistent sagittal plane underloading during rehabilitation following ACLR. However, as most available evidence remains observational, further longitudinal and interventional studies are required to determine whether modifying these biomechanical alterations improves long-term structural and clinical outcomes. Furthermore, a stiff gait has been associated with reduced shock absorption and altered joint contact mechanics, which may further influence long-term joint health [88]. These findings highlight the potential clinical importance of identifying and addressing persistent biomechanical asymmetries during rehabilitation. However, longitudinal and interventional research is required to determine whether modifying these gait adaptations leads to improved clinical outcomes or reduced osteoarthritis risk.

### Study Limitations

This meta-analysis has several limitations that should be acknowledged. As with any systematic review, the findings may be influenced by potential sources of bias, including possible publication bias and limitations related to the search strategy, despite adherence to PRISMA guidelines. Although Egger’s regression did not identify statistically significant funnel plot asymmetry, publication bias cannot be definitively excluded, as statistical tests for funnel plot asymmetry have limited power and primarily detect small-study effects rather than true publication bias.

Most notably, considerable statistical heterogeneity was observed across several meta-analyses, which may have influenced the precision and generalizability of pooled estimates. Although most included studies reported participants’ sex, biomechanical outcomes were rarely reported separately for males and females, preventing meaningful sex-specific analyses. Moreover, several studies included mixed-sex cohorts without clearly reporting sex distribution. This limitation is important because previous research has demonstrated sex-specific biomechanical characteristics following ACLR. For instance, Asaeda et al. [45], identified sex differences in tibial rotation during the stance phase of gait, both pre-operatively and post-ACLR. Future research should specifically focus on the reporting of sex-specific data to close the sex data gap. Authors of the included studies did not adequately describe the graft type that was used during surgical treatment which is why we were unable to compute graft-specific analyses. Nevertheless, we cannot rule out that the graft type has an impact on knee stability and mobility. In fact, Webster et al. [41] showed that the graft type (hamstring or patellar tendon) impacted on gait characteristics of ACLR patients., While gait speed is a relevant marker of mobility, we were unable to conduct gait speed specific analyses due to a lack of data reported in the included studies. Only cross-sectional studies were eligible to be included in this meta-analysis which examined biomechanical differences across timepoints post-ACLR. However, longitudinal studies following patients at different time points following ACLR are needed to fully understand how gait biomechanics change over time, and how these changes may influence the development of OA or re-injury. Including multiple independent subgroups from a single study may have increased the relative weighting of that study’s population within pooled analyses. The review focused exclusively on walking biomechanics, and higher-demand tasks such as running or jumping may reveal more pronounced deficits.

## Conclusions

This meta-analysis included 31 studies and demonstrated that individuals following ACLR exhibit persistent alterations in walking biomechanics compared with healthy controls. Specifically, ACLR patients demonstrated lower peak knee flexion angles and reduced sagittal plane knee joint moments during the stance phase of gait. These findings collectively suggest a walking pattern characterized by a straighter knee posture and reduced dynamic loading of the lower limb, which appears to persist for at least 12 months following surgery. The consistent observation of reduced sagittal plane knee loading suggests a potential underloading strategy that may reflect protective neuromuscular adaptations, quadriceps dysfunction, altered motor control, or behavioral factors such as fear-avoidance. Although such adaptations may initially reduce mechanical joint stress, persistent alterations in joint loading patterns may influence load distribution across articular structures and could have implications for long-term joint health. Although these biomechanical alterations are consistently observed, the direct clinical consequences remain incompletely understood. Future longitudinal studies are required to determine whether these gait adaptations are associated with long-term outcomes such as persistent symptoms, reinjury risk, or the development of post-traumatic osteoarthritis. Furthermore, interventional studies are necessary to evaluate whether targeted rehabilitation strategies can modify these biomechanical asymmetries and whether such modifications lead to improved clinical or structural outcomes.

## Supplementary Information

Supplementary Material 1.

## Data Availability

The datasets used and/or analyzed during the current study available from the corresponding author on reasonable request.

## Abbreviations

ACL: Anterior cruciate ligament
ACLR: ACL reconstruction
PEDro: Physiotherapy Evidence Database
CENTRAL: Cochrane Central Register of Controlled Trials
SMD: Standardized mean differences
CI: Confidence intervals
Std: Standardized
PRISMA: Preferred Reporting Items for Systematic Reviews and Meta-Analyses
PECOS: (Participants, exposure, comparators, outcomes, and study design)
ROM: Range of motion
2D: 2-dimensional
3D: 3-dimensional
NR: Not reported
SD: Standard deviation, M,male
F: Female
HQ: High quality
MQ: Moderate quality
LQ: Low quality
HT: Hamstring tendon graft
PT: Patellar tendon graft
A: Early ACLR
B: Mid ACLR
C: Late ACLR
HIGH: High body mass index
LOW: Low body mass index
PT-Q: Partial-thickness graft harvests
FT-Q: Full-thickness graft harvests
AMP: Anteromedial portal technique
TT: Traditional transtibial reconstruction
