# Supplementary material for: Time-Dependent Alterations in Walking Biomechanics After Anterior Cruciate Ligament Reconstruction Compared with Healthy Individuals: A Systematic Review with Meta-analysis
Source: Sports Med Open. 2026 Apr 6;12:39. doi: 10.1186/s40798-026-01016-x (PMC13053734; doi:10.1186/s40798-026-01016-x)
Supplement: Supplementary file 1 — Supplementary Material 1. [file 40798_2026_1016_MOESM1_ESM.docx]

**Appendix 1: Search syntax for different electronic databases**

Table S1: Search syntax for PubMed from inception until February 2025

| Search number | Query |
| --- | --- |
| 15 | #2 AND #8 AND #14 |
| 14 | #11 OR #12 OR #13 |
| 13 | "Lower Extremity"[Mesh] OR "Lower Extremity Deformities, Congenital"[Mesh] OR "Foot"[Mesh] "Foot Joints"[Mesh] OR "Ankle Joint"[Mesh] OR "Ankle"[Mesh] OR "Knee Joint"[Mesh] OR "Knee"[Mesh] OR "Hip Joint"[Mesh] OR "Hip"[Mesh] OR "Pelvis"[Mesh] OR "Thigh"[Mesh] |
| 12 | "Lower limb*" [ti] OR "lower extremit*" [ti] OR foot [ti] OR feet [ti] OR ankle [ti] OR ankles [ti] OR leg [ti] OR legs [ti] OR knee [ti] OR knee* [ti] OR hip [ti] OR hips [ti] OR pelvis [ti] OR thigh [ti] OR thighs [ti] |
| 11 | "Lower limb*" [tiab] OR "lower extremit*" [tiab] OR foot [tiab] OR feet [tiab] OR ankle [tiab] OR ankles [tiab] OR leg [tiab] OR legs [tiab] OR knee [tiab] OR knee* [tiab] OR hip [tiab] OR hips [tiab] OR pelvis [tiab] OR thigh [tiab] OR thighs [tiab] |
| 9 | #4 AND #8 |
| 8 | #5 OR #6 OR #7 |
| 7 | biomechanic* [ti] OR kinematic* [ti] OR motion* [ti] OR movement* [ti] OR pressure* [ti] OR dynamic [ti] OR load* [ti] OR biomech* [ti] OR mechanic* [ti] OR shock* [ti] OR absorb* [ti] OR friction* [ti] OR moment* [ti] OR angle* [ti] OR rotation* [ti] OR force* [ti] OR "angular impuls*" [ti] OR velocit* [ti] OR speed* [ti] OR acceleration* [ti] OR muscle* [ti] OR activit* [ti] OR torque* [ti] OR power* [ti] |
| 6 | (friction* [tiab] OR moment* [tiab] OR angle* [tiab] OR rotation* [tiab] OR force* [tiab] OR angular* [tiab] Or impuls* [tiab] OR velocit* [tiab] OR speed* [tiab] OR acceleration* [tiab] OR activit* [tiab] OR mechanic* [tiab] OR power* [tiab] OR biomechanic* [tiab] OR kinematic* [tiab] OR motion* [tiab] OR movement* [tiab] OR pressure* [tiab] OR dynamic* [tiab] OR load* [tiab] OR biomech* [tiab] OR mechanic* [tiab] OR shock* [tiab] OR absorb* [tiab]) |
| 5 | "biomechanical phenomena"[Mesh] OR "mechanical phenomena"[Mesh] |
| 4 | #1 OR #2 OR #3 |
| 3 | “anterior cruciate ligament reconstruction” [tiab] OR "anterior cruciate ligament" [tiab] OR "anterior cruciate ligament injur*" [tiab] OR “ACL repair” [tiab] |
| 2 | "anterior cruciate ligament" [ti] OR "anterior cruciate ligament injur*" [ti] OR “ACL repair” [ti] |
| 1 | "anterior Cruciate Ligament"[Mesh] OR "anterior cruciate ligament reconstruction"[Mesh] OR "anterior cruciate ligament injur*"[Mesh] |

Search results based on PubMed 598

Table S2: Search syntax for Physiotherapy Evidence Database (PEDro) from inception until February 2025

| First search | Second search | Third search |
| --- | --- | --- |
| • Abstract & title: anterior cruciate ligament* AND | • Abstract & title: anterior cruciate ligament* AND | • Abstract & title: anterior cruciate ligament* AND |
| • Body part: foot and ankle AND | • Body part: lower leg and knee AND | • Body part: thigh or hip AND |
| • Method: clinical trial | • Method: clinical trial | • Method: clinical trial |

Search results based on PEDro: 346

Table S3: Search syntax for Scopus from inception until February, 2025 (**1,284** results)

| Participants | AND | Task | AND | Outcomes | AND | Body part |
| --- | --- | --- | --- | --- | --- | --- |
| "anterior cruciate ligament reconstruction" |  | walk* |  | biomech* |  | "lower limb*" |
| OR "anterior cruciate ligament" |  | OR walking* |  | OR kinetic* |  | OR "lower extremit*" |
| OR "anterior cruciate ligament injur*" |  | OR gait* |  | OR kinematic* |  | OR foot |
| OR "ACL repair" |  | OR "translation movement" |  | OR speed |  | OR feet |
|  |  |  |  | OR force* |  | OR ankle |
|  |  |  |  | OR motion* |  | OR ankles |
|  |  |  |  | OR rotation* |  | OR leg |
|  |  |  |  | OR impuls |  | OR legs |
|  |  |  |  | OR acceleration |  | OR knee |
|  |  |  |  | OR dynamic |  | OR knee* |
|  |  |  |  | OR power |  | OR hip |
|  |  |  |  | OR movement |  | OR hips |
|  |  |  |  | OR load* |  | OR pelvis |
|  |  |  |  | OR joint moment* |  | OR thigh* |
|  |  |  |  | OR ground reaction force* |  |  |
|  |  |  |  | OR mechanic* |  |  |
|  |  |  |  | OR torque* |  |  |
|  |  |  |  | OR angle* |  |  |

**1,284** results from Scopus

Table S4: Search syntax for Web of Science from inception until February, 2025

| Participants | AND | Task | AND | Outcomes | AND | Body part |
| --- | --- | --- | --- | --- | --- | --- |
| "anterior cruciate ligament reconstruction" |  | walk* |  | biomech* |  | "lower limb*" |
| OR "anterior cruciate ligament" |  | OR walking* |  | OR kinetic* |  | OR "lower extremit*" |
| OR "anterior cruciate ligament injur*" |  | OR gait* |  | OR kinematic* |  | OR foot |
| OR "ACL repair" |  | OR "translation movement" |  | OR speed |  | OR feet |
|  |  |  |  | OR force* |  | OR ankle |
|  |  |  |  | OR motion* |  | OR ankles |
|  |  |  |  | OR rotation* |  | OR leg |
|  |  |  |  | OR impuls |  | OR legs |
|  |  |  |  | OR acceleration |  | OR knee |
|  |  |  |  | OR dynamic |  | OR knee* |
|  |  |  |  | OR power |  | OR hip |
|  |  |  |  | OR movement |  | OR hips |
|  |  |  |  | OR load* |  | OR pelvis |
|  |  |  |  | OR joint moment* |  | OR thigh* |
|  |  |  |  | OR ground reaction force* |  |  |
|  |  |  |  | OR mechanic* |  |  |
|  |  |  |  | OR torque* |  |  |
|  |  |  |  | OR angle* |  |  |

**809** results from Web of Science core collection

Table S5: Search syntax for Cochrane Central Register of Controlled Trials (central) from inception until February 2025

| ID | Search |
| --- | --- |
| #1 | MeSH descriptor: [anterior cruciate ligament] explode all trees |
| #2 | MeSH descriptor: [biomechanical phenomena] explode all trees |
| #3 | biomech* |
| #4 | kinetic* |
| #5 | kinematic* |
| #6 | Speed |
| #7 | force* |
| #8 | motion* |
| #9 | rotation* |
| #10 | Impulse |
| #11 | Acceleration |
| #12 | Dynamic |
| #13 | Power |
| #14 | Movement |
| #15 | load* |
| #16 | joint moment* |
| #17 | ground reaction force* |
| #18 | mechanic* |
| #19 | torque* |
| #20 | angle* |
| #21 | #2 OR #3 OR #4 OR #5 OR #6 OR #7 OR #8 OR #9 OR #10 OR #11 OR #12 OR #13 OR #14 OR #15 OR #16 OR #17 OR #18 OR #19 OR #20 |
| #22 | MeSH descriptor: [Lower Extremity] explode all trees |
| #23 | #1 AND #21 AND #22 |

Total of 39 hits for Cochrane Central Register

**Appendix 2: Risk of Bias Assessment: Modified Downs and Black Checklist**

Table S6. Modified Downs and Black items used in this review

| Item No. | Assessment Item (Modified Wording) | Domain | Scoring Criteria |
| --- | --- | --- | --- |
| 1 | Is the study objective, hypothesis, or aim clearly described? | Reporting | Yes = 1; No = 0 |
| 2 | Are the main outcome measures clearly described in the Introduction or Methods section? | Reporting | Yes = 1; No = 0 |
| 3 | Are participant characteristics clearly described (e.g., inclusion/exclusion criteria, case definition, source of controls)? | Reporting | Yes = 1; No = 0 |
| 4 | Are the interventions or conditions of interest clearly described? | Reporting | Yes = 1; No = 0 |
| 5 | Are the distributions of principal confounders clearly described for each comparison group? | Reporting / Confounding | Yes = 2; Partially = 1; No = 0 |
| 6 | Are the main findings clearly reported with sufficient outcome data to support conclusions? | Reporting | Yes = 1; No = 0 |
| 7 | Are estimates of random variability provided for the main outcomes (e.g., SD, SE, CI, or IQR)? | Reporting | Yes = 1; No = 0 |
| 10 | Are exact probability values reported (e.g., p = 0.035 rather than p < 0.05), except when p < 0.001? | Reporting | Yes = 1; No = 0 |
| 11 | Were recruited participants representative of the target source population? | External Validity | Yes = 1; No / Unable to determine = 0 |
| 12 | Were participants who agreed to participate representative of the entire eligible population? | External Validity | Yes = 1; No / Unable to determine = 0 |
| 15 | Was blinding implemented for individuals measuring primary outcomes? | Internal Validity — Bias | Yes = 1; No / Unable to determine = 0 |
| 16 | Were unplanned or post-hoc analyses clearly identified (i.e., data dredging transparently reported)? | Internal Validity — Bias | Yes = 1; No / Unable to determine = 0 |
| 18 | Were appropriate statistical tests used for the main outcomes? | Internal Validity — Bias | Yes = 1; No = 0 |
| 20 | Were outcome measures valid and reliable? | Internal Validity — Bias | Yes = 1; No = 0 |
| 21 | Were comparison groups recruited from the same population? | Internal Validity — Confounding (Selection Bias) | Yes = 1; No / Unable to determine = 0 |
| 22 | Were participants in comparison groups recruited during the same time period? | Internal Validity — Confounding (Selection Bias) | Yes = 1; No / Unable to determine = 0 |
| 25 | Was adequate adjustment made for confounding variables in statistical analyses? | Internal Validity — Confounding (Selection Bias) | Yes = 1; No = 0 |
| 27 | Did the study report sufficient statistical power or sample size justification to detect clinically important effects? | Power | Yes = 1; No = 0 |

(Original Downs and Black Checklist is 27 items; items irrelevant to biomechanics intervention-free designs were removed or modified).

Table S7. Operational Definitions for Each Item

| Item No. | Operational Definition Used in This Review |
| --- | --- |
| Reporting Domain |  |
| 1 | The study explicitly states a clear hypothesis, aim, or objective related to the investigated biomechanical or clinical outcomes. |
| 2 | Primary outcomes are clearly defined in the Introduction or Methods section. Studies introducing primary outcomes only in the Results section were scored negatively. |
| 3 | Participant characteristics are adequately described, including demographic information, inclusion/exclusion criteria, case definitions, and source of control groups when applicable. |
| 4 | Interventions or conditions of interest are clearly described, including experimental tasks, comparison groups, and treatment or surgical procedures where relevant. |
| 5 | Principal confounders relevant to outcome interpretation are reported and described for comparison groups. Full reporting was scored as “Yes,” partial reporting as “Partially,” and absence as “No.” |
| 6 | Main findings are clearly presented with sufficient numerical outcome data to allow interpretation of results and verification of conclusions. |
| 7 | Measures of random variability are reported for main outcomes, including standard deviation, standard error, confidence intervals, or interquartile ranges. |
| 10 | Exact probability values are reported for primary outcomes, except where p-values are smaller than 0.001. |
| External Validity Domain |  |
| 11 | The recruited study sample is representative of the source population, with the sampling frame and recruitment procedures clearly described. |
| 12 | Participants who agreed to participate are representative of the eligible population, with recruitment rates or selection procedures described. |
| Internal Validity – Bias Domain |  |
| 15 | Attempts were made to blind assessors measuring primary outcomes or to reduce measurement bias through standardized procedures. |
| 16 | The study clearly states whether analyses were pre-planned. Post-hoc or exploratory analyses were identified when applicable. |
| 18 | Statistical analyses used to evaluate main outcomes are appropriate for study design, sample size, and data distribution. |
| 20 | Outcome measurement tools are valid, reliable, and clearly described, or supported by previous validation studies. |
| Internal Validity – Confounding (Selection Bias) Domain |  |
| 21 | Comparison groups were recruited from the same source population or clinical setting. |
| 22 | Comparison groups were recruited during the same time period or recruitment timeframe was adequately controlled. |
| 25 | Analyses appropriately controlled or adjusted for relevant confounding variables influencing primary outcomes. |
| Power Domain |  |
| 27 | Studies reported sample size calculation, power analysis, or included a sample size considered sufficient to detect clinically meaningful effects. |

**Rationale for Using the Modified Downs and Black Checklist**

The modified Downs and Black Checklist was selected because:

1. **It is suitable for non-randomized biomechanical studies**

Most included studies were cross-sectional or observational between-participant comparisons of ACLR versus healthy control groups. The original Downs and Black tool, widely used in physiotherapy, sports medicine, and biomechanics systematic reviews, is designed to assess reporting quality, external validity, internal validity (bias and confounding), and statistical power without assuming an interventional design.

1. **Modified Downs and Black tool allows domain-level mapping**

Items in the Downs and Black Quality Index tool included domains such as confounding, selection, measurement of outcomes, and reporting. This allows transparent reporting while preserving methodological appropriateness for biomechanical studies.

1. **Consistency with prior gait-biomechanics systematic reviews**

Recent biomechanical systematic reviews have used the Downs and Black tool or modified variants, making it a field-standard tool for ACL gait research.

Appendix 3: Funnel plots

**Figure S1.** Funnel plot indicating potential publication bias for the parameter peak knee flexion angle.

**Figure S2.** Funnel plot indicating potential publication bias for the parameter peak knee adduction angle.

**Figure S3.** Funnel plot indicating potential publication bias for the parameter peak hip flexion angle.

** Figure S4.** Funnel plot indicating potential publication bias for the parameter peak vertical ground reaction force.

**Figure S5.** Funnel plot indicating potential publication bias for the parameter peak knee flexion moment.

**Figure S6.** Funnel plot indicating potential publication bias for the parameter peak knee extension moment.

**Figure S7.** Funnel plot indicating potential publication bias for the parameter peak knee adduction moment.

**Figure S8.** Funnel plot indicating potential publication bias for the parameter peak hip flexion moment.
